# Reductions in midbrain GABAergic and dopamine neuron markers are linked in schizophrenia

**DOI:** 10.1186/s13041-021-00805-7

**Published:** 2021-06-26

**Authors:** Tertia D. Purves-Tyson, Amelia M. Brown, Christin Weissleder, Debora A. Rothmond, Cynthia Shannon Weickert

**Affiliations:** 1 Schizophrenia Research Laboratory, Neuroscience Research Australia, 139 Barker Street, Margarete Ainsworth Building, Level 5, Randwick, NSW 2031 Australia; 2 School of Psychiatry, Faculty of Medicine, University of New South Wales, Sydney, NSW 2052 Australia; 3 Department of Neuroscience & Physiology, Upstate Medical University, Syracuse, NY 13210 USA

**Keywords:** GABA, GABRA, GAD1, GAD65/67, Parvalbumin, Somatostatin, Tyrosine hydroxylase, Substantia nigra, Schizophrenia, Neuroinflammation

## Abstract

Reductions in the GABAergic neurotransmitter system exist across multiple brain regions in schizophrenia and encompass both pre- and postsynaptic components. While reduced midbrain GABAergic inhibitory neurotransmission may contribute to the hyperdopaminergia thought to underpin psychosis in schizophrenia, molecular changes consistent with this have not been reported. We hypothesised that reduced GABA-related molecular markers would be found in the midbrain of people with schizophrenia and that these would correlate with dopaminergic molecular changes. We hypothesised that downregulation of inhibitory neuron markers would be exacerbated in schizophrenia cases with high levels of neuroinflammation. Eight GABAergic-related transcripts were measured with quantitative PCR, and glutamate decarboxylase (GAD) 65/67 and GABA_A_ alpha 3 (α3) (GABRA3) protein were measured with immunoblotting, in post-mortem midbrain (28/28 and 28/26 control/schizophrenia cases for mRNA and protein, respectively), and analysed by both diagnosis and inflammatory subgroups (as previously defined by higher levels of four pro-inflammatory cytokine transcripts). We found reductions (21 – 44%) in mRNA encoding both presynaptic and postsynaptic proteins, vesicular GABA transporter (*VGAT*), *GAD1*, and parvalbumin (*PV*) mRNAs and four alpha subunits (α1, α2, α3, α5) of the GABA_A_ receptor in people with schizophrenia compared to controls (*p* < 0.05). Gene expression of somatostatin (*SST*) was unchanged (*p* = 0.485). We confirmed the reduction in GAD at the protein level (34%, *p* < 0.05). When stratifying by inflammation, only *GABRA3* mRNA exhibited more pronounced changes in high compared to low inflammatory subgroups in schizophrenia. GABRA3 protein was expressed by 98% of tyrosine hydroxylase-positive neurons and was 23% lower in schizophrenia, though this did not reach statistical significance (*p* > 0.05). Expression of transcripts for GABA_A_ receptor alpha subunits 2 and 3 (*GABRA2*, *GABRA3*) were positively correlated with tyrosine hydroxylase (TH) and dopamine transporter (DAT) transcripts in schizophrenia cases (*GABRA2; r* > 0.630, *GABRA3; r* > 0.762, all *p* < 0.001) but not controls (*GABRA2; r* < − 0.200, *GABRA3; r* < 0.310, all *p* > 0.05). Taken together, our results support a profound disruption to inhibitory neurotransmission in the substantia nigra regardless of inflammatory status, which provides a potential mechanism for disinhibition of nigrostriatal dopamine neurotransmission.

**Supplementary Information:**

The online version contains supplementary material available at 10.1186/s13041-021-00805-7.

## Introduction

Dopamine (DA) dysregulation, which can be traced to a common neural origin in the midbrain, manifests in striatal hyperdopaminergia, which is linked to psychosis, and cortical hypodopaminergia, which contributes to cognitive deficits [1, 2]. Importantly, gamma aminobutyric acid (GABA) neurotransmission controls activity of dopamine neurons within the midbrain [3–6], yet the status of the midbrain GABA neurons has not been thoroughly examined in schizophrenia. Among the most consistently replicated neurobiological changes in schizophrenia is a reduction in GABAergic transmitter system markers. Reductions in gene and protein expression of GABAergic inhibitory interneuron markers and other GABA-related molecules in schizophrenia are demonstrated across multiple brain regions, including cortex, hippocampus, striatum and cerebellum, pointing to widespread deficits in inhibitory neurotransmission [7–11]. Thus, we sought to determine whether deficits in inhibitory neurotransmission extend to the midbrain in schizophrenia.

Within the midbrain, the substantia nigra (SN) is the origin of the dopaminergic nigrostriatal projections to the dorsal striatum. The dorsal striatum [12–14] and the SN [1, 2, 14–18] are identified as major loci of dopamine dysregulation in schizophrenia. We have previously reported dysregulation of multiple DA-related mRNAs in the midbrain in schizophrenia, including lower gene expression of DA receptor D2 isoforms (*DRD2short*, and *DRD2long*), DA transporter (*DAT*), vesicular monoamine transporter (*VMAT*), and a synthesis enzyme (*aromatic acid decarboxylase*) [17]. The SN consists of the predominantly dopaminergic pars compacta (SNpc), and the predominantly GABAergic pars reticulata (SNpr) [19, 20]. SNpc dopamine neurons receive excitatory input from the cortex and thalamus, and inhibitory input both from GABAergic projection neurons in the striatum and SNpr, and interneurons in the SNpc [21]. A loss of GABAergic inhibitory control at the site of origin of dopamine neurons may lead to increased dopamine activity [22]. Here, we sought to test if molecular deficiencies in GABAergic inhibitory interneurons occur in the midbrain of people with schizophrenia.

GABAergic neurons are identified by expression of the GABA synthesising enzyme glutamate decarboxylase (GAD) isoforms, GAD67 (*GAD1*) and GAD65 (*GAD2*), expressed by all interneuron subtypes [23, 24]. A reduction in cortical GAD67 mRNA and protein is consistently demonstrated in schizophrenia [25–29]. However, contrary to our prediction of a deficit in inhibition, a single study reports elevated GAD67 protein in the midbrain in schizophrenia [30]. Changes in GAD are often, but not always [31], accompanied by alterations in vesicular GABA transporter (VGAT) [27, 32] and in other inhibitory interneuron markers. GABAergic inhibitory interneuron subtypes are identified by expression of neuropeptides and calcium-binding proteins [33–35]. In schizophrenia, gene and protein expression of interneuron markers, particularly parvalbumin (PV) and somatostatin (SST), are often reduced in the cortex [7, 28, 32, 36–39], cerebellum [9, 10], and hippocampus [8]. Interestingly, PV + cells found within the SN are not morphologically consistent with interneurons [34], rather local inhibitory control in the SN is from axon collaterals of PV-expressing projection neurons that have their cell bodies in the SNpr and project axons to the thalamus or superior colliculus [3, 21, 34, 40, 41]. Branches of these axons synapse directly onto the dopaminergic neurons of the SNpc [42, 43]. Within the SNpc, 30% of neurons express *GAD1* mRNA [44], and immunohistochemical and in situ hybridisation studies have identified SST-positive neurons in the midbrain [45, 46]. It is unknown whether there are molecular changes in *GAD1*, *PV* and *SST* transcripts in the midbrain in schizophrenia.

Neuroinflammation is implicated in the pathophysiology of schizophrenia and is linked to inhibitory interneuron deficits in the cortex [47–53]. We recently identified heightened neuroinflammation in the midbrain of people with schizophrenia [54, 55]. Gene expression of inflammatory markers, particularly *IL1B*, *IL6*, *SERPINA3* (and *TNFA* in midbrain), are used to identify schizophrenia cases with ‘low’ and ‘high’ inflammatory biotypes [50, 51, 54, 56, 57]. In the cortex, schizophrenia cases with a high inflammatory biotype have lower *GAD1, SST*, and *PV* mRNAs when compared to schizophrenia cases with the low inflammatory biotype [50]. Furthermore, increased gene expression of the viral restriction factor interferon-induced transmembrane protein (*IFITM*) is linked to lower expression of GABA-related mRNAs, including *GAD1* and *SST*, in the cortex of schizophrenia cases [53]. We therefore hypothesised that changes in GABA synthesis and interneuron marker gene expression would be exacerbated in the midbrain of schizophrenia cases with a high inflammatory biotype.

Regulation of GABAergic neurotransmission also occurs at the level of postsynaptic receptors and alterations in GABA_A_ subunits are identified in schizophrenia [58, 59]. The ionotropic GABA_A_ receptor has multiple subunits that differ both functionally and in their regional specificity [60, 61]. In the SN, the α1 subunit is expressed almost exclusively in the SNpr, and α2 and α5 are expressed in both the SNpc and SNpr, albeit at relatively low levels [62–69]. GABA_A_ receptors that express the α3 subunit are highly expressed on dopaminergic neurons of the SNpc [63, 64, 69], though, to our knowledge, this subunit has not been explored in schizophrenia. In schizophrenia, gene expression of GABA_A_ receptor alpha subunits one (α1), two (α2), and five (α5) are altered in dorsolateral prefrontal cortex and hippocampus [7, 8, 25], but the levels of these subunit mRNAs are unexplored in the midbrain. We hypothesised that transcripts encoding the alpha subunits of the GABA_A_ receptors would be expressed at lower levels in the midbrain in schizophrenia and that these reductions would be exacerbated in schizophrenia cases with a high inflammatory biotype.

We previously showed lower gene expression of some mRNAs encoding proteins involved in dopamine neurotransmission in the post-mortem midbrain in schizophrenia [17]. To further elucidate whether and how changes in the GABAergic system in the midbrain may contribute to dopamine dysregulation in schizophrenia, we explored the relationships between GABA- and DA-related transcripts in controls and schizophrenia cases, to identify whether these relationships are altered in the disease state.

To test our hypotheses, we sought to determine in a human midbrain cohort (28/28 and 28/26 control/schizophrenia cases for mRNA and protein, respectively), whether *GAD1* mRNA and GAD protein, as well as *PV*, *SST* and *VGAT* mRNA transcripts are lower in the midbrain in schizophrenia. We sought to determine relative levels of *GABRA1*, *GABRA2*, *GABRA3*, and *GABRA5* mRNAs, GABRA3 protein levels, and to determine cellular location of GABRA3 protein in the midbrain in schizophrenia. Furthermore, we sought to determine whether any measured alterations in GABA-related transcripts were related to a heightened state of neuroinflammation in the midbrain. We also investigated the relationships between GABA-related transcripts and DA-related transcripts.

## Methods

### Post-mortem midbrain tissue and cohort demographics

Midbrain tissue was sourced from the NSW Brain Tissue Resource Centre and experiments were approved by the University of New South Wales Human Research Ethics Committee (HREC #17826). The tissue samples (30 schizophrenia, 30 controls) were dissected from 60 µm cryostat sections of the midbrain cut in the coronal plane at the level of the oculomotor nerve exit. The area excised included both the SNpc and SNpr, found in the ventral mesencephalon below the red nucleus, as previously described [17]. RNA was extracted from six 60 µm tissue sections for each case.

Cases in both the mRNA and protein cohorts were matched on age, sex, and post-mortem interval (PMI), as previously described [17] (Table 1). pH was significantly lower in schizophrenia patients compared to controls. RNA integrity number (RIN), age, and PMI did not change significantly between schizophrenia cases and control subjects (Table 1), or between inflammatory subgroup (Table 2). The average RIN of control cases was 5.56 ± 1.15 (range 3.0–7.3), and 5.61 ± 1.31 (range 3.2–8.3) for schizophrenia cases (Table 1). Samples with RINs < 3.0 were excluded from further analysis. The RIN cut-off was selected to retain as many cases as possible. Samples with lower RIN values can produce accurate data so long as this is factored into the statistical analysis (refer to Statistical Analysis) [70, 71]. We use the geometric mean of four housekeeping mRNAs as a normaliser for our genes of interest to account for any degradation of transcripts at different levels of mRNA abundance, and this denominator serves as an index of RNA degradation and is accounted for in our analysis [72]. The final midbrain mRNA cohort comprised 28 schizophrenia cases and 28 controls as 3 cases (1 control, 2 schizophrenia) were excluded due to RINs < 3.0, and 1 control case due to insufficient cDNA amplification. The final protein cohort consisted of 26 schizophrenia cases and 28 controls. This midbrain mRNA cohort has previously been divided into low and high inflammatory biotype, as determined through a recursive two-step cluster analysis of gene expression of *SERPINA3*, *IL6*, *IL1B*, and *TNFA* (detailed in [54]). No control cases had a high inflammatory biotype, whilst 13 schizophrenia cases had a high inflammatory biotype, and the remaining 15 were classified with a low inflammatory biotype (Table 2) [54]. The cases retained in the mRNA and protein cohorts do not completely overlap, as 2–4 different cases per group were excluded from each cohort due to poor quality protein and/or mRNA/cDNA amplification. In addition, not all cases included in the protein cohort have an assigned inflammatory biotype due to poor RNA/insufficient cDNA amplification.

**Table 1:** Demographic details of the post-mortem midbrain mRNA and protein cohorts classified by diagnosis. Table previously published in [17, 55]

Demographics	mRNA Cohort			Protein Cohort		
	Control (28)	SCZ (28)	Statistics	Control (28)	SCZ (26)	Statistics
Age (years) (range)	50.54 (22–67)	51.36 (26–67)	t(54) = − 0.27, *p* = 0.79	52.21 (22–69)	52.29 (26—67)	t(52) = − 0.23, *p* = 0.82
Sex (M,F)	(20,8)	(19,9)	χ^2^(1,56) = 0.084, *p* = 0.771	(19,9)	(16,10)	χ^2^(1,54) = 0.236, *p* = 0.627
pH	6.66 ± 0.26	6.51 ± 0.20	*t(54)* = *2.52, p* = 0.015*	6.69 ± 0.24	6.51 ± 0.23	*t(52)* = *2.62, p* = 0.011*
PMI (hours) (range)	31.68 ± 10.21 (15–50)	35.66 ± 17.71 (5–72)	t(54) = − 1.03, *p* = 0.31	32.75 ± 9.90 (15–50)	38.21 ± 18.20 (5–72)	t(52) = − 1.38, *p* = 0.17
RIN (range)	5.56 ± 1.15 (3.0–7.3)	5.61 ± 1.31 (3.2–8.3)	t(54) = − 0.15, *p* = 0.88	–	–	–
Duration of illness	–	28.31 ± 12.72 (4–49)	–	–	29.12 ± 13.02 (4–49)	–
Daily CPZ (mg)	–	736.45 ± 520.50	–	–	716.37 ± 557.76	–
Last recorded CPZ (mg)	–	597.54 ± 497.64	–	–	616.20 ± 506.60	–
Lifetime CPZ (g)	–	8231.44 ± 8714.24	–	–	8427.92 ± 9348.47	–

Bold text indicates statistically significant data

*F* female, *M* male, *PMI* post-mortem interval, *RIN* RNA integrity, *SCZ* schizophrenia, *CPZ* chlorpromazine equivalent

Data are mean ± s.d. Age and PMI ranges are in brackets. Note the group sizes are different in the cohorts as different cases were excluded with either poor quality of RNA and/or protein. A schizophrenia case in this mRNA cohort was lost from the mRNA cohort published previously [17] due to failure of cDNA synthesis

* *p* < 0.05

**Table 2:** Demographic details of the post-mortem midbrain mRNA and protein cohorts classified by inflammatory subgroup. Table previously published in [54]

Demo-graphics	mRNA Cohort				Protein Cohort			
	Control (28)	SCZ low inflammatory (15)	SCZ high inflammatory (13)	Statistics	Control (26)^	SCZ low inflammatory (13)^	**SCZ high inflammatory (12)**	**Statistics**
Age (years) (range)	50.54 (22–67)	48.27 (30–64)	54.92 (26–67)	H(2) = 2.81, p = 0.25	51.19 (22–67)	49.31 (30–64)	56.17 (26–67)	H(2) = 2.89, p = 0.24
Sex (M,F)	(20,8)	(11,4)	(8,5)	χ^2^(2,56) = 0.543, *p* = 0.762	(19,7)	(9,4)	(7,5)	χ^2^(2,51) = 0.832, *p* = 0.660
pH	6.66 ± 0.26	6.52 ± 0.20	6.49 ± 0.20	*H(2)* = *8.51, p* = 0.014*	6.69 ± 0.22	6.54 ± 0.20	6.52 ± 0.18	F(2,48) = 4.02, *p* = 0.024*
PMI (hours) (range)	31.68 ± 10.21 (15–50)	33.43 ± 14.66 (18–64)	38.23 ± 21.01 (5–72)	F(2,53) = 0.91, *p* = 0.41	33.42 ± 10.21 (15–50)	35.19 ± 14.93 (18–64)	39.62 ± 21.30 (5–72)	F(2,48) = 1.00, *p* = 0.38
RIN (range)	5.56 ± 1.15 (3.0–7.3)	5.53 ± 1.28 (3.3–7.2)	5.70 ± 1.39 (3.2–8.3)	F(2,53) = 0.079, p = 0.92	–	–	–	–
Duration of illness	–	26.43 ± 11.65	31.62 ± 13.926	t(26) = − 1.073, p = 0.293	–	25.92 ± 11.30	32.33 ± 14.29	t(22) = − 1.220, *p* = 0.24
Daily CPZ (mg)	–	483.54 ± 177.63	1039.93 ± 637.06	*t(10.169)* = *2.68, p* = 0.023*	–	422.30 ± 116.23	1043.11 ± 675.69	*U* = *81.0, p* = 0.002***
Last recorded CPZ (mg)	–	361.53 ± 316.56	869.85 ± 538.82	*U* = *156.5, p* = 0.005**	–	391.00 ± 331.35	860.17 ± 561.60	*U* = *210.5, p* = 0.019*
Lifetime CPZ (g)	–	4204.952 ± 2170.41	13,063.22 ± 11,129.88	*U* = *100.0, p* = 0.007**	–	3799.83 ± 1800.43	13,570.25 ± 11,681.88	*U* = *77.0, p* = 0.008**

Bold text indicates statistically significant data

*F* female, *M* male, *PMI* post-mortem interval, *RIN* RNA integrity, *SCZ* schizophrenia, *CPZ* chlorpromazine equivalent

Data are mean ± s.d. Age and PMI ranges are in brackets. ^group sizes are different in the Protein Cohort inflammatory subgroups as we were unable to determine inflammatory biotype for some cases due to poor RNA quality/insufficient amplification of cDNA

* *p* < 0.05, ** *p* < 0.01, *** *p* < 0.001

All schizophrenia cases were treated with antipsychotics, the calculated doses of which were standardised, as previously described [17], to a chlorpromazine equivalent (CPZ) incorporating measures of lifetime, daily dosage, and last dosage. Other ante-mortem factors, including illness duration, smoking (lifetime and at time of death), the nature of symptoms (positive or negative, and depressive), agonal state, and suicide as the cause of death, were recorded. Additional information about freezer storage time, antipsychotic types, alcohol use, toxicology screening, alcohol consumption and history of depression are included in Additional file 1.

### RNA extraction and quantitative real-time PCR

Total RNA was extracted from midbrain samples using TRIzol (Invitrogen, Mulgrave, VIC, AUS). Tissue was homogenised in 800 µl of TRIzol using a disposable tissue pestle (Axygen, Mount Martha, VIC, Australia) and incubated at room temperature for 5 min. Chloroform (160µL) was added, and the mixture was vortexed, then centrifuged for 15 min (12,000*g* at 4 °C). The aqueous phase was transferred to a new tube. 400 µl of isopropanol was added and incubated for 10 min, and the mixture spun for 10 min (12,000*g* at 4 °C). Isopropanol was removed, and the pellet was washed in 800µL of 70% ethanol, and then centrifuged for 5 min (7500*g* at 4 °C). Ethanol was removed; samples were air-dried and resuspended in 30 µl RNAse-free water (Sigma-Aldrich, Castle Hill, NSW, AUS). RNA was quantified by nanodrop using a ND-1000 Spectrophotometer (Nanodrop Technologies, Wilmington, DE, USA). RNA integrity number (RIN) was measured for each sample using Agilent Bioanalyzer 2100 (Agilent Technologies, Santa Clara, CA, USA). Complementary DNA (cDNA) was generated using Superscript IV First Strand Synthesis Kit and random hexamers (18091200; Invitrogen) according to the manufacturer’s protocol.

TaqMan gene expression assays (Invitrogen) (Additional file 1: Table S1) were used to run high-throughput qPCR (Fluidigm; Ramaciotti Centre for Genomics, UNSW, Sydney) for seven genes, and a single qPCR for *GABRA3* was run on an Applied Biosystems Prism 7900HT Fast Real Time system. In addition, we re-measured tyrosine hydroxylase *(TH*) and *DAT* mRNAs using the same TaqMan gene expression assays as previously [17] but with the high-throughput Fluidigm qPCR method. PCR cycling conditions were 50 °C for 2 min, 95 °C for 10 min, then 50 cycles of 95 °C for 15 s and 60 °C for 1 min. Samples in the single qPCR were run in triplicate, samples measured by Fluidigm were run in singulate. Serial dilutions of pooled cDNA from all samples were used to quantitate sample expression by the relative standard curve method. To confirm replicability between the qPCR methods, and the use of triplicates or singulates, we show that relative gene expression of DAT and β-actin measured using both methods are highly significantly positively correlated (*DAT* mRNA, N = 53, R^2^ = 0.893, *p* < 0.0001; *β-actin* mRNA, N = 55, R^2^ = 0.680, *p* < 0.0001).

Gene expression of eight GABAergic markers was measured: *GAD1*, *PV*, *SST*, *VGAT, GABRA1*, *GABRA2*, *GABRA3*, and *GABRA5*. Gene expression data was analysed with Fluidigm Real-Time PCR Analysis software (version 4.5.2), or SDS software (version 2.4; ABI, Life Technologies). Gene expression was normalised to the geomean of four housekeeping genes measured by high-throughput qPCR: β-actin, TATA-box binding protein, ubiquitin-C, and glyceraldehyde 3-phosphate dehydrogenase. No differences were found either between the expression of housekeeping genes by diagnosis, nor in the geomean across experimental groups (all t < 1.160, df = 53–54, *p* > 0.05). Gene expression data are presented as relative mRNA levels ± SEM.

### Western blotting

Protein was extracted as previously described [17]. In brief, samples were homogenised in 0.1 M Tris (pH 7.5), 50% glycerol, protease inhibitor cocktail and aprotinin (0.015 mM) (all Sigma-Aldrich) with a handheld electric homogeniser (Polytron, Kinematica, Lucerne, Switzerland). Protein was quantified using a Bradford protein assay (B6916, Sigma-Aldrich). For each case, 5 μg (for GAD) and 6 μg (for GABRA3) protein was diluted in NuPAGE Sample Buffer (NP0007) and NuPAGE Sample Reducing Agent (NP0009) and separated on a NuPAGE Tris–Acetate 3–8% gel (WG1603A). Samples were run alongside Precision Plus Protein Standard (Biorad, Gladesville, NSW, AUS, #161–0374) and an internal control (IC) comprised of randomly selected samples pooled from within the cohort (70 min, 150 V). Three gels were required to run the full midbrain cohort and each gel had an IC to allow comparison between immunoblots. Samples were distributed randomly across the three gels. Proteins were transferred onto nitrocellulose membranes with the Power Blotter System (PB0012; all reagents Invitrogen). Membranes were rinsed in tris-buffered saline with Tween-20 (TBST) and blocked for 2 h in 5% skim milk in TBST. GAD protein was detected with a rabbit anti-glutamate decarboxylase 65/67 antibody (1:1000, AB1511, Sigma-Aldrich), previously used to show expected and specific expression in the cell bodies and processes of interstitial white matter neurons in the cortex [73], and GABRA3 protein with rabbit anti-GABA Receptor α3 antibody (1:1000, ab224214, Abcam, Melbourne, VIC, AUS). Primary antibodies were diluted in 1% skim milk in TBST and incubated overnight at 4 °C. Membranes were rinsed with TBST and incubated with a horseradish peroxidase-conjugated (HRP) secondary antibody (1:5000, AP307P, Merck-Millipore, Bayswater, VIC, AUS) for 1 h at room temperature. Membranes were rinsed and re-probed with mouse anti-β-actin antibody (1:5000, MAB1501, Merck-Millipore), incubated at room temperature for at least 2 h, then incubated with HRP-conjugated goat anti-mouse secondary antibody for 1 h (1:5000, AP124P, Merck-Millipore). Full Western blots are presented in Additional file 1: Fig. S2. The GABRA3 Western blot shown was stripped with 2 × 15 min washes in 0.025 M glycine, 1.5% SDS buffer prior to being re-blocked and re-probed with the β-actin secondary antibody. The GAD Western Blot shown was not stripped.

Protein bands were visualised with high-sensitivity enhanced chemiluminescent substrate (WBKLS0100, Merck-Millipore), and imaged on the iBright 1500 Imaging System (Invitrogen) to provide a density measurement for each band. For GAD65/67, a single band was detected at ~ 66 kDa. A prominent ~ 55 kDa band was detected for GABRA3. On each separate Western blot, for the IC, the band density of the protein of interest was divided by the band density of β-actin to generate a normalised IC value for each blot. Each sample on the Western blot was then divided by this value such that the normalised IC value on each separate Western blot is equivalent to 1 and band densities of samples can then be compared across multiple Western blots in a single experiment. As such, the band density of the protein of interest for each sample was divided by the density of the ~ 42 kDa β-actin band for the same sample (to give a normalised value), and this value was divided by the normalised IC value for that immunoblot to give a measure of relative protein expression. The band density of β-actin did not differ according to diagnosis [t(46–53) < 0.4782, p > 0.635].

### Immunohistochemistry

Double-label immunofluorescence for GABRA3 and TH was performed to confirm GABRA3 expression by dopaminergic neurons using 14 μm fresh frozen midbrain tissue sections from 3 control and 3 high inflammatory schizophrenia cases. Sections were fixed in 4% PFA (Sigma-Aldrich) (10 min, 4 °C) and blocked for 1 h in 10% normal donkey serum (Millipore-Merck), 0.3% Triton X-100 (Sigma-Aldrich) and 0.05% BSA in PBS for 1 h. Slides were incubated overnight at 4 °C with rabbit anti-GABRA3 (1:200, ab224214, Abcam) and mouse anti-TH (1:400, MAB318, Millipore-Merck) antibodies prepared in 0.3% Triton X-100 and 0.05% BSA in PBS. Slides were washed 3 × 10 min in PBS and Alexa Fluor-conjugated secondary antibodies (AF594 anti-rabbit; AF488 anti-mouse, both 1:500, Life Technologies, AUS) were added for 1 h at room temperature. To minimise auto-fluorescence, slides were washed in 15 mM cupric sulphate (Sigma-Aldrich) and 50 mM ammonium acetate (Sigma-Aldrich) for 2 × 15 min [74]. Slides were counterstained with DAPI (1:1000, Sigma-Aldrich) in PBS and cover-slipped with anti-fade mounting media (Citifluor anti-fadent, ProSciTech, Kirwan, QLD, Australia). A negative control slide without primary antibodies was included and did not produce any signal (Additional file 1: Fig. S1).

Images of GABRA3 and TH double-labelling immunohistochemistry were taken on a LSM800 Zeiss confocal microscope (Zeiss Australia, Lonsdale, SA, AUS), equipped with a high efficiency GaAsP detector, two multi-alkali photomultiplier tubes and the following objectives: 10× air objective with numerical aperture (NA) 0.45, 20× air objective with NA 0.8 and 40× oil objective with NA 1.3. Detection wavelengths were 490–580 nm for AF488, 580–700 nm for AF594 and 400–580 nm for DAPI. For quantification of the percentage of TH^+^ neurons expressing GABRA3, three z-stacks of each case were acquired using the 10 × air objective [0.7 zoom, average thickness µm ± standard deviation (range): 13.80 ± 2.60 (10.62–19.47)]. Maximum intensity projections were used to visualise the brightest voxel in the final image. TH^+^ neurons with abundant immunostaining in the soma were counted (75 ± 30 per case, 451 total TH^+^ neurons) and the percentage of TH^+^ cells co-expressing GABRA3 was determined. Additional z-stacks were acquired using the 20× air and 40× oil objectives [average thickness µm ± standard deviation (range): 8.12 ± 3.38 (5.2–12.96)]. Image analysis and quantification were conducted using Zeiss Zen software version 3.1 (blue edition, Zeiss, Australia) and ImageJ version 1.50e (NIH, Bethesda, MD, USA).

### Statistical Analysis

SPSS was used for all statistical analysis (IBM SPSS Statistics, version 25) and significance was set at the level of *p* < 0.05. Outliers, identified as values beyond two standard deviations of the mean, were excluded from subsequent analysis. Data for each gene or protein of interest was tested for normality using the Shapiro–Wilk test, and homogeneity of variance using Levene’s test. *SST*, *PV* mRNA and GAD protein did not pass Levene’s test. All transcripts except *GABRA1* and *PV* were normally distributed when considered by diagnosis, when considered by diagnosis/inflammatory subgroup, all transcripts except *PV* were normally distributed.

#### Covariates

For each transcript or protein of interest, the relationship to PMI, pH, and age was assessed by Pearson’s correlations (Additional file 1: Table S2). RIN was assessed as a covariate for transcripts of interest. GAD and GABRA3 protein were not correlated with PMI, pH, or age. Variables that correlated with gene expression were incorporated as covariates in an analysis of covariance (ANCOVA). No gene expression was significantly correlated with age, and PMI was significantly correlated only with *PV* mRNA. RIN was frequently correlated with GABAergic gene expression. Brain pH was correlated with gene expression of multiple GABA-related transcripts (Additional file 1: Table S2), but not used as a covariate, as lower pH levels are considered to be part of the disease process in schizophrenia [75], and co-varying for pH may inappropriately “correct” for variance due to disease.

#### Analysis of relative gene expression by diagnosis and by diagnosis/inflammatory subgroup

For genes and proteins of interest that were normally distributed by diagnosis, an independent samples two-tailed *t* test was used if data was not correlated with any demographic variables. ANCOVAs were run for transcripts that correlated with demographic variables. *GABRA1* and *PV* mRNA levels could not be transformed to normality, and these variables were ranked and residualised by the rank of their covariates. *GABRA1* mRNA levels were tested with a Mann–Whitney U test, and *PV* mRNA with an independent samples two-tailed *t*-test (*PV* mRNA levels were normally distributed after residualisation, whereas *GABRA1* mRNA levels were not).

For analysis by diagnosis/inflammatory subgroups, data was analysed with ANOVA or ANCOVA, where appropriate. *GABRA1* mRNA levels underwent a square root transformation of the inflammatory subgroups to reach normality and ranked *PV* mRNA was residualised with ranked PMI and RIN to reach normality. Where significant effects were observed, the analysis was followed by a least significant difference (LSD) *post-hoc* test.

#### Correlations between GABA- and DA-related gene expression

Correlations between DA- and GABA-related gene expression were investigated in control and schizophrenia groups separately. The dopaminergic markers included were *TH* and *DAT* mRNAs (both log transformed to normality), which were examined for correlations between each of *GABRA1*, *GABRA2*, *GABRA3*, and *GABRA5*. Pearson’s correlations were run to assess covariates with age, PMI, and RIN in both the dopaminergic and GABAergic genes. If a covariate was present in either marker, partial correlations were run. To determine if correlation coefficients in control and schizophrenia groups were significantly different, they were converted to *z* scores using Fisher's *r-*to-*z* transformation.

#### Analysis of pre- and ante-mortem factors and antipsychotic treatment

The genes of interest were statistically explored to consider alterations due to clinical and ante-mortem variables. Spearman’s correlations were used to explore the relationships between antipsychotic treatment measures as well as duration of illness (in years), and transcripts of interest (Additional file 1: Table S3). Age was used as a covariate in the correlation between *SST* mRNA and duration of illness, as SST levels decrease with age [36, 38]. Other clinical variables measured were antipsychotic type (first-generation, second-generation, or both), clozapine use (indicative of treatment resistance), and whether the patient exhibited predominantly positive or negative symptoms (Additional file 1). Ante-mortem variables explored included lifetime smoking, and lifetime symptoms of depression, as well as variables assessed at the time of death—smoking at time of death, agonal state, and suicide (Additional file 1). Clinical variables were assessed with independent samples two-tailed *t* tests or one-way ANOVA. Suicide was only reported as the cause of death for some schizophrenia cases and no controls and was assessed by independent samples two-tailed *t* tests. All other variables were assessed by two-way ANOVA with diagnosis and the variable of interest as independent variables. Results are considered exploratory due to small and uneven group sizes, and assumptions of normality and homogeneity of variance were not met.

## Results

### GAD gene and protein expression in the midbrain were lower in schizophrenia, but not different according to inflammatory status

**Fig. 1:**
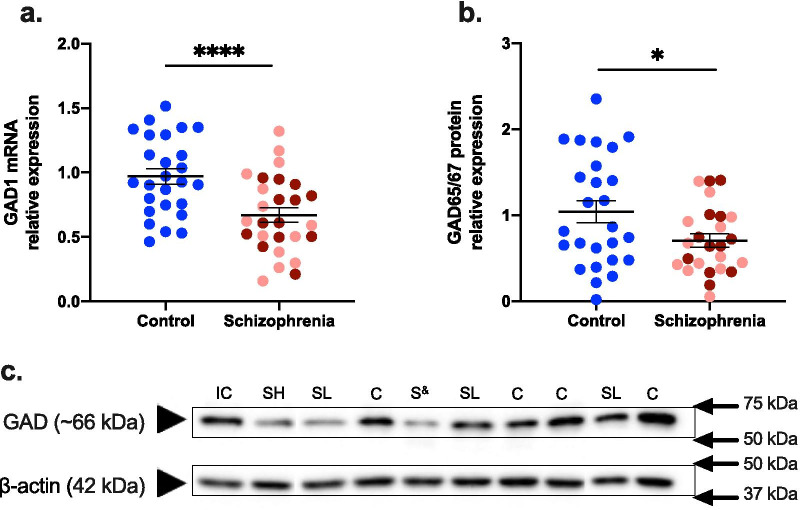
Gene (GAD1) and protein (GAD65/67) expression levels of glutamate decarboxylase (GAD) in the midbrain in schizophrenia. **a** GAD1 mRNA was 30.96% lower in schizophrenia cases relative to controls, and this was not exacerbated by inflammatory status. b GAD65/67 protein was 34.14% lower in schizophrenia cases compared to controls, and this was also independent of inflammatory status. Pink dots are low inflammatory/schizophrenia cases, dark red dots are high inflammatory/schizophrenia cases. c A GAD65/67 protein band (~ 66 kDa) was detected in all control and schizophrenia cases. β-actin (42 kDa) was used as a loading control.* IC* internal control, *C* control, *SH* schizophrenia high inflammatory, *SL* schizophrenia low inflammatory. ^&^ inflammatory status unknown. Statistical results for analysis by inflammatory subgroup are in Table 3. Bars indicate mean ± SEM. *p < 0.05, ****p < 0.0001

As hypothesised, expression of GAD was lower at both mRNA and protein levels in the midbrain in schizophrenia. When analysed by diagnosis, midbrain *GAD1* gene expression was highly significantly decreased (30.96%) when schizophrenia cases were compared to controls (F(1,50) = 15.142; *p* < 0.0001) (Fig. 1a). In contrast to our hypothesis, deficits in *GAD1* mRNA were not exacerbated in the high inflammatory/schizophrenia subgroup (Table 3). Rather, *GAD1* mRNA was expressed at lower and comparable levels in both inflammatory subgroups, 31.88% in the high inflammatory/schizophrenia subgroup and 30.10% in the low inflammatory/schizophrenia subgroup, compared to the control group (F(2,49) = 7.551; *p* = 0.001, both schizophrenia-control comparisons *p* < 0.001) (Table 3). *GAD1* mRNA levels did not differ between the low and high inflammatory/schizophrenia subgroups (*p* = 0.655) (Table 3).

**Table 3:** Statistical results for mRNA and protein levels when analysed by inflammatory subgroup

Marker	Overall ANOVA	CTRL low vs SCZ low	CTRL low vs SCZ high	SCZ low vs SCZ high
*GAD1* mRNA	F(2,49) = 7.551; p = 0.001**	30.10%; *p* < 0.001***	31.88%; *p* < 0.001***	*p* = 0.655
GAD65/67 protein	F(2,46) = 2.895, *p* = 0.065			
*PV* mRNA	F(2,51) = 2.751, *p* = 0.073			
*SST* mRNA	F(2,49) = 1.859, *p* = 0.167			
*VGAT* mRNA	F(2,52) = 8.281, *p* = 0.001**	28.66%; *p* = 0.018*	43.94%; *p* < 0.001***	*p* = 0.177
*GABRA1* mRNA	F(2,51) = 9.221, *p* < 0.0001****	34.91%, *p* = 0.004**	44.85%, *p* < 0.0001****	*p* = 0.368
*GABRA2* mRNA	F(2,50) = 2.605, *p* = 0.084			
*GABRA3* mRNA	F(2,50) = 9.788; *p* = 0.0001****	*p* = 0.181	35.68%, *p* < 0.0001****	27.72%, *p* = 0.007**
GABRA3 protein	F(2,40) = 1.157, p = 0.325			
*GABRA5* mRNA	F(2,49) = 1.555, *p* = 0.221			

Percentage reduction and result of post-hoc tests are reported when result of overall ANOVA was significant. Significant results are bolded

* *p* < 0.05, ** *p* < 0.01, *** *p* < 0.001, **** *p* < 0.0001

Western blotting revealed an anti-GAD65/67 immunoreactive band at the expected size (~ 66 kDa) in all human midbrain cases (Fig. 1c) (a full Western Blot is shown in Additional file 1: Fig. S2A). Similar to *GAD1* mRNA, when analysed by diagnosis, GAD65/67 protein expression was significantly decreased by 34.14% in the midbrain of schizophrenia cases compared to controls (t(41.286) = 2.406, *p* = 0.021) (Fig. 1b). Although GAD65/67 protein expression was 30.05% and 36.73% lower in the midbrain in the high inflammatory and low inflammatory/schizophrenia subgroups, respectively, compared to controls, this did not reach statistical significance overall when analysed by inflammatory subgroup (F(2,46) = 2.895, *p* = 0.065) (Table 3).

### *PV* and *VGAT* mRNAs were decreased in the midbrain in schizophrenia, whereas *SST* mRNA was unchanged

**Fig. 2:**
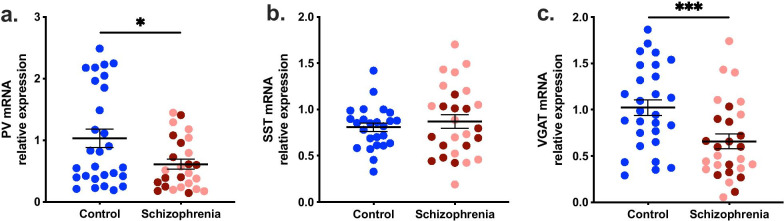
Gene expression level of interneuron markers and vesicular GABA transporter in control and schizophrenia midbrain. **a** PV mRNA was lower in the midbrain of schizophrenia cases (by 40.70%) relative to controls and this was not exacerbated by inflammatory status. **b** SST mRNA did not show significant changes in the midbrain between schizophrenia cases and controls. **c** VGAT mRNA was 39.69% lower in schizophrenia cases compared with controls and this was also not exacerbated by inflammatory status. Pink dots are low inflammatory/schizophrenia cases, dark red dots are high inflammatory/schizophrenia cases. Statistical results for analysis by inflammatory subgroup are in Table 3. Bars indicate mean ± SEM. *p < 0.05, ***p < 0.001

As predicted, *PV* mRNA was decreased by 40.70% in the midbrain of schizophrenia cases relative to controls (t(52) = 2.349, *p* = 0.023) (Fig. 2a) and this decrease was independent of inflammatory status (Table 3). Contrary to our hypothesis, *SST* mRNA was unchanged in the midbrain of schizophrenia cases compared to controls (t(43.071) = -0.705, *p* = 0.485) (Fig. 2b) or when analysed by inflammatory subgroup (F(2,49) = 1.859, *p* = 0.167) (Table 3).

*VGAT* mRNA was decreased (39.69%) in the midbrain of schizophrenia cases relative to controls (F(1,52) = 17.733; *p* = 0.0001) (Fig. 2c). Analysis by inflammatory subgroup (F(2,52) = 8.281, *p* = 0.001) indicated that this decrease was due to reductions in both the inflammatory/schizophrenia subgroups (28.66%, low inflammatory, *p* = 0.018; 43.94%, high inflammatory, *p* < 0.0001), and no significant difference between the two schizophrenia inflammatory subgroups was identified (*p* = 0.177) (Table 3).

### Four GABA alpha subunit mRNAs were lower in the midbrain in schizophrenia, with only *GABRA3* mRNA reductions related to neuroinflammation

**Fig. 3:**
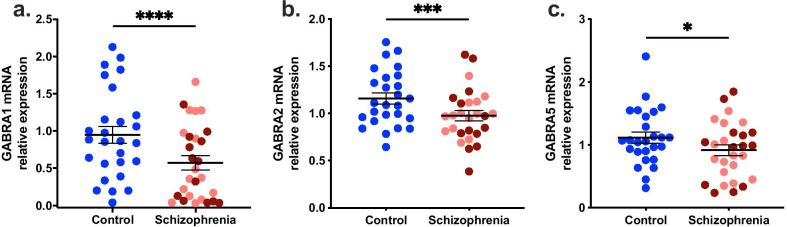
GABA_A_ receptor subunit gene expression in the midbrain in schizophrenia. **a** GABRA1 mRNA was 43.77% lower in the midbrain from schizophrenia cases compared with controls. **b** GABRA2 mRNA was 21.53% lower in the midbrain from schizophrenia cases compared with controls. **c** GABRA5 mRNA showed a decrease of 20.98% in the midbrain of schizophrenia cases compared with controls. Pink dots are low inflammatory/schizophrenia cases, dark red dots are high inflammatory/schizophrenia cases. Reductions in GABRA1, 2 and 5 mRNA levels were not exacerbated by neuroinflammation. Statistical results for analysis by inflammatory subgroup are in Table 3. Bars indicate mean ± SEM. *p < 0.05, ***p < 0.001, ****p < 0.0001

Gene expression of GABA_A_ receptor alpha 1, 2, 3 and 5 subunits were significantly decreased in the midbrain of schizophrenia cases compared with controls. *GABRA1* mRNA was 43.77% lower in the midbrain of schizophrenia cases compared with controls (U = 156.0; *p* < 0.0001) (Fig. 3a). *GABRA1* mRNA was significantly changed when analysed by inflammatory subgroup (F(2,51) = 9.221, *p* < 0.0001), however, whilst the control group was significantly different to both the low inflammatory (34.91%, *p* = 0.004) and high inflammatory (44.85%, *p* < 0.0001) schizophrenia subgroups, there was no significant difference between the low and high inflammatory/schizophrenia subgroups (*p* = 0.368) (Table 3).

*GABRA2* mRNA was decreased by 21.53% in the midbrain in schizophrenia cases compared to controls (t(50) = 3.369; *p* = 0.001) (Fig. 3b) and was unchanged when analysed by inflammatory subgroup (F(2,50) = 2.605, *p* = 0.084) (Table 3). There was a 20.98% decrease in *GABRA5* mRNA (F(1,50) = 4.913; *p* = 0.031; Fig. 3c) in the midbrain in schizophrenia cases relative to controls. *GABRA5* mRNA levels were unchanged when comparing the high versus low inflammatory schizophrenia subgroups (Table 3).

**Fig. 4:**
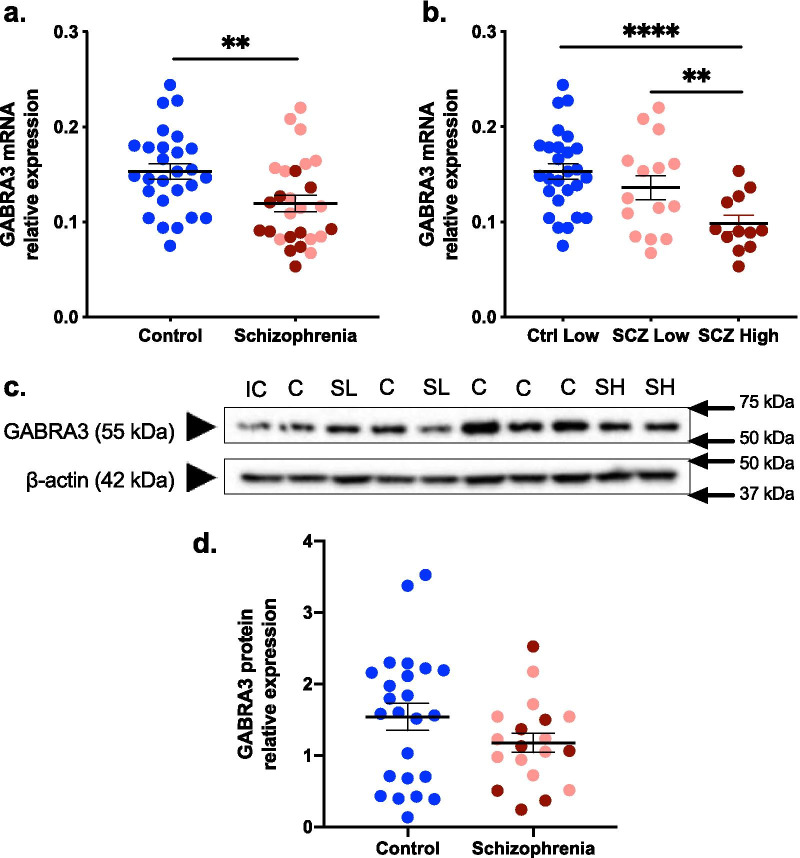
GABA_A_ alpha 3 subunit gene expression in the midbrain of control and schizophrenia cases. **a** GABRA3 mRNA was 21.98% lower in patients with schizophrenia relative to controls. **b** Reductions in GABRA3 mRNA were exacerbated in the high inflammatory/schizophrenia subgroup. GABRA3 mRNA was lower in high inflammatory/schizophrenia cases compared with both low inflammatory/schizophrenia cases and controls. There was no difference in GABRA3 mRNA between low inflammatory/schizophrenia cases compared with controls. **c** A GABA_A_ α3 subunit protein band was detected at ~ 55 kDa in human midbrain by an antibody raised against amino acids 6–125. β-actin (42 kDa) was used as a loading control. **d** GABRA3 protein was 23.24% lower in schizophrenia cases relative to controls, though this change did not reach statistical significance. Pink dots are low inflammatory/schizophrenia cases, dark red dots are high inflammatory/schizophrenia cases. *SCZ* schizophrenia, *IC* internal control, *C* control, *SH* schizophrenia high inflammatory, *SL* schizophrenia low inflammatory. Statistical results for analysis by inflammatory subgroup are also in Table 3. Bars indicate mean ± SEM. **p < 0.01., ****p < 0.0001

By diagnosis, *GABRA3* mRNA was significantly decreased by 21.98% in the midbrain in schizophrenia cases compared with controls (F(1,51) = 10.128, *p* = 0.002) (Fig. 4a). When analysed by inflammatory subgroup, lower levels of *GABRA3* mRNA in the midbrain were only found in the high inflammatory/schizophrenia subgroup. Levels of *GABRA3* mRNA (F(2,50) = 9.788; *p* < 0.0001) were significantly lower, by 35.68% in the high inflammatory/schizophrenia subgroup compared to controls (*p* < 0.0001) and by 27.72% in the high inflammatory/schizophrenia subgroup compared to the low inflammatory/schizophrenia subgroup (*p* = 0.007) (Fig. 4b) (Table 3). *GABRA3* mRNA in the low inflammatory/schizophrenia subgroup was not significantly different to the control group (*p* = 0.181).

Western blotting revealed an expected prominent GABRA3 protein band of ~ 55kDA (Fig. 4c) (a full Western Blot is shown in Additional file 1: Fig. S2B). Diagnostically, GABRA3 protein levels were 23.24% lower in schizophrenia, however this change did not reach statistical significance (t(45) = 1.519, p = 0.136) (Fig. 4d). GABRA3 protein levels were unchanged by inflammatory subgroup (F(2,40) = 1.157, p = 0.325) (Table 3).

**Fig. 5:**
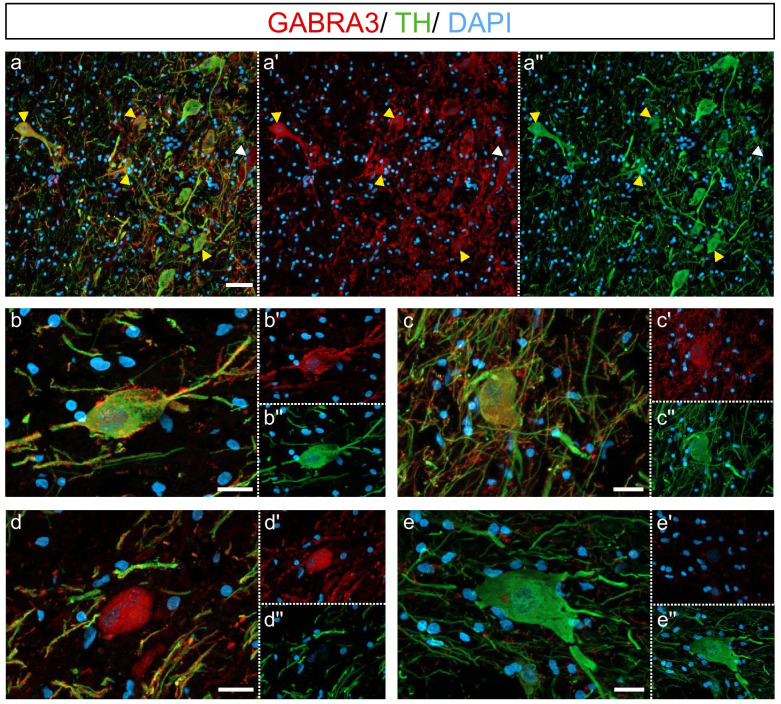
GABA_A_ alpha 3 subunit expression was abundant in dopaminergic neurons in the midbrain. **a** Double-labelling immunohistochemistry revealed GABRA3^+^/TH^+^ neurons with variable levels of GABRA3 (yellow arrowheads) and GABRA3^+^/TH^−^ cells (white arrowhead) in the substantia nigra. GABRA3 expression was identified along the cell membrane and processes (**b**) and in the cytoplasm of TH^+^ neurons (**c**). GABRA3^+^/TH^−^ cells (**d**) and GABRA3^−^/TH^+^ neurons (**e**) were occasionally identified in the midbrain in schizophrenia and controls cases. Scale bars = 50 μm (**a**) and 20 μm (**b**–**e**)

Double-labelling immunohistochemistry revealed GABRA3 expression often overlapped with TH expression irrespective of diagnostic groups (Fig. 5a). GABRA3 was often expressed along the cell membrane (Fig. 5b) and in the cytoplasm of TH^+^ neurons (Fig. 5c). When counting double-labelled GABRA3^+^/TH^+^ and single-labelled GABRA3^−^/TH^+^ neurons in 3 control and 3 high inflammatory/schizophrenia cases, we identified GABRA3 expression in 98% of TH^+^ neurons, irrespective of diagnostic group. In addition to the abundant co-localisation of GABRA3 and TH, we identified some single-labelled GABRA3^+^/TH^−^ cells (Fig. 5d) and the occasional GABRA3^−^/TH^+^ cell in the SN (Fig. 5e).

### *GABRA2* and *GABRA3* mRNAs were strongly positively associated with *TH* and *DAT* mRNAs in schizophrenia

**Fig. 6:**
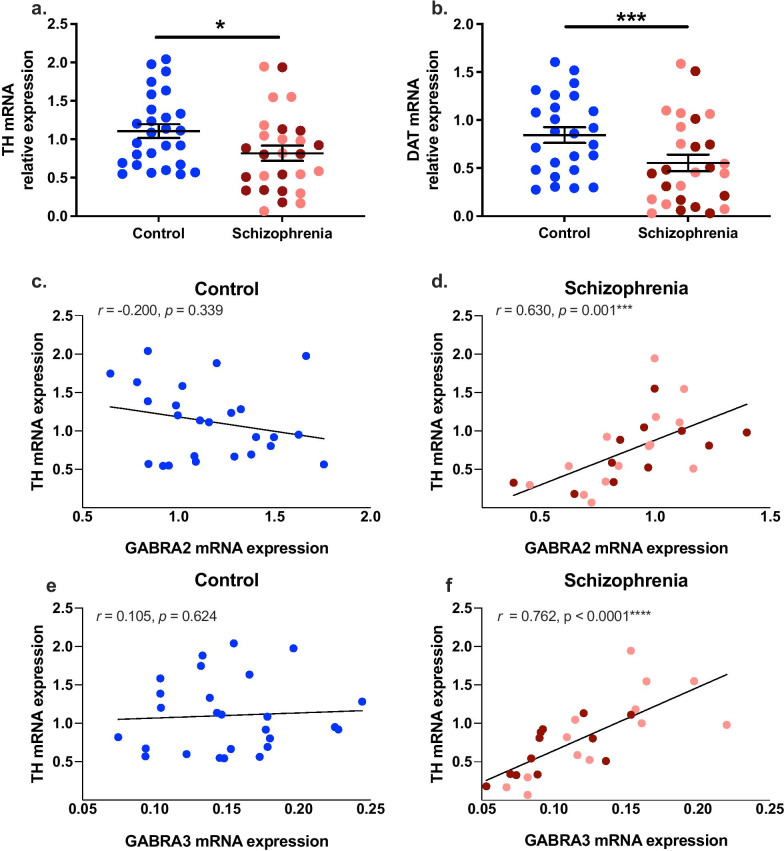
TH and DAT gene expression in the midbrain of controls and schizophrenia cases, and the relationships between TH and GABRA2 and GABRA3 mRNAs in the midbrain in control and schizophrenia cases. **a** TH mRNA and **b** DAT mRNA levels in the midbrain were significantly lower in the schizophrenia group relative to the controls. **c** TH and GABRA2 mRNAs were not correlated in controls, **d** but were strongly positively correlated in schizophrenia cases. The strength of these correlations was significantly different (z = − 3.13, p = 0.002) . Similarly, TH and GABRA3 mRNA were not significantly correlated in controls, **e** but were strongly positively correlated in schizophrenia cases (**f**). The strength of these correlations was significantly different (z = − 3.04, p = 0.002). Pink dots are low inflammatory/schizophrenia cases, dark red dots are high inflammatory/schizophrenia cases. Bars indicate mean ± SEM. *p < 0.05., ***p < 0.001

In contrast to previously reported, whereby lower TH mRNA levels in the schizophrenia cases did not reach statistical significance [17], *TH* mRNA was significantly lower in the schizophrenia group compared to the control group *t*(52) = 2.175, p = 0.012 (Fig. 6a). Similar to previously reported [17], *DAT* mRNA was significantly lower in the schizophrenia group compared to controls [F(1,50) = 10.306, p = 0.002 (RIN)] (Fig. 6b).

*GABRA2* (*r* = 0.630, *p* = 0.001), and *GABRA3* (*r* = 0.762, *p* < 0.0001) mRNAs were significantly positively correlated with *TH* mRNA in schizophrenia cases, but not in control cases (*r* = -0.200, *p* = 0.339; *r* = 0.105, *p* = 0.624, respectively), and these correlations were significantly different when comparing diagnostic groups (*GABRA2*: z = -3.13, *p* = 0.002 and *GABRA3*: z = -3.04, *p* = 0.002) (Fig. 6c–f, Table 4).

**Table 4:** Correlations between GABAergic transcripts and TH and DAT mRNAs in control and schizophrenia cases. Differences between the correlation coefficients were assessed with Fisher’s r-to-z transformation

Transcripts	CONTROL			SCHIZOPHRENIA			Fisher´s r-to-z transformation	
	r	p	n	r	p	n	z	p
TH								
GABRA1	0.159	0.447	26	**0.456**	0.022*	**26**	− 1.13	0.259
GABRA2	− 0.200	0.339	25	**0.630**	0.001***	**25**	− **3.13**	0.002**
GABRA3	0.105	0.624	26	**0.762**	< 0.0001****	**26**	− **3.04**	0.002**
GABRA5	0.304	0.140	25	0.250	0.228	26	0.20	0.842
DAT								
GABRA1	0.336	0.093	27	**0.526**	0.008**	**25**	− 0.80	0.424
GABRA2	− 0.030	0.887	26	**0.790**	< 0.0001****	**24**	− **3.65**	< 0.001***
GABRA3	0.310	0.132	27	**0.847**	< 0.0001****	**25**	− **3.13**	0.017*
GABRA5	**0.472**	0.017*	**26**	0.320	0.127	25	0.61	0.542

Bold text indicates statistically significant data

* *p* < 0.05, ** *p* < 0.01, *** *p* < 0.005, **** *p* < 0.0001

Similar relationships were evident between these two subunits and *DAT* mRNA (Table 4). *GABRA2* (*r* = 0.790, *p* < 0.0001), and *GABRA3* (*r* = 0.847, *p* < 0.0001) mRNAs were significantly positively correlated with *DAT* mRNA in schizophrenia cases, but not in control cases (*r* = -0.030, *p* = 0.887; *r* = 0.310, *p* = 0.132, respectively), and these correlations were significantly different when comparing diagnostic groups (*GABRA2*: z = -3.65, *p* < 0.001 and *GABRA3*: z = -3.13, *p* = 0.017) (Table 4).

*GABRA1* mRNA was not significantly correlated with *TH* and *DAT* mRNAs in controls (both: *r* < 0.336, *p* > 0.05) but was positively correlated with TH and DAT mRNAs in schizophrenia cases (both: *r* > 0.456, *p* < 0.05) (Table 4). *GABRA5* mRNA was significantly positively correlated with *DAT* mRNA (*r* = 0.472, *p* = 0.017) but not *TH* mRNA (*r* = 0.304, *p* = 0.140) in controls cases and was not correlated with *TH* or *DAT* mRNAs in schizophrenia cases (both: *r* < 0.320, *p* > 0.05) (Table 4). However, neither *GABRA1* or *GABRA5* mRNAs exhibited significantly changed relationships with *TH* or *DAT* mRNAs between control and schizophrenia (all: z < 1.13, *p* > 0.05) (Table 4).

### Relationship of genes of interest to clinical and ante-mortem variables

No measures of antipsychotic treatment (last, mean daily, and lifetime chlorpromazine equivalent dose) were significantly correlated with expression of any gene of interest (Additional file 1: Table S3). *GAD1* mRNA levels were significantly higher in schizophrenia patients that died by suicide compared to schizophrenia cases that died by non-suicide causes (t(25) = -2.071, *p* = 0.049) (Additional file 1: Table S4). GAD protein levels were significantly positively correlated with duration of illness (*r* = 0.490, *p* = 0.015) (Additional file 1: Table S3). No genes of interest differed by agonal state, smoking, antipsychotic type, symptom category (positive vs negative) or severity (treatment resistance), or lifetime depression status (Additional file 1: Table S4, all *p* > 0.05).

## Discussion

We found pronounced deficits in transcripts and proteins involved in GABA synthesis, vesicular packaging and signal transduction in the ventral midbrain in schizophrenia compared to controls. Contrary to our prediction, these reductions in GABAergic markers, including *GAD* mRNA and protein, *VGAT*, *PV*, *GABRA1*, *GABRA2*, and *GABRA5* mRNAs, with the exception of *GABRA3* mRNA, were not exacerbated by neuroinflammation. Interestingly, the unexpected positive relationship detected between DA transcripts and some GABA receptor transcripts was contrary to the predicted negative relationship [22], and indicates an aberrant relationship between these two neurotransmitter systems at the level of the midbrain in schizophrenia. These molecular changes related to midbrain inhibitory neurotransmission appear to be intrinsic to the disease state, as they are generally not exacerbated by a higher inflammatory state nor indeed directly correlated with antipsychotic usage. This study is the first to provide evidence for blunted GABAergic neurotransmission in the post-mortem ventral midbrain in schizophrenia.

The reduction in gene expression of the GABA synthesis enzyme, *GAD1*, suggests that there is less production of GABA within neuron cell bodies in the midbrain in schizophrenia. This is also reflected in lower GAD protein expression, although protein measurements incorporate GAD synthesised within the midbrain, as well as GAD expressed by terminals projecting to the midbrain and synthesised elsewhere [76]. Our report of lower *VGAT* mRNA expression supports that there may be less inhibition in the midbrain in schizophrenia, and aligns with a study indicating that less GAD activity decreases VGAT activity [77]. In conjunction with lower *VGAT* mRNA, suggesting that less packaging of GABA into the synaptic vesicles of these neurons may occur, our results suggest that there may be less GABA released into the synaptic cleft to bind to diminished postsynaptic GABA receptors and thus less inhibitory action within the midbrain. This is supported by an electron microscopy study that reported a decrease in inhibitory synapses in the midbrain in schizophrenia (8 controls, 11 schizophrenia cases) [78]. Our findings in the midbrain concord with the widespread reduction of GAD mRNA and protein across multiple brain regions in schizophrenia [25–29], although they are in contrast to a single immunoblotting study in the midbrain demonstrating increased GAD67 protein in schizophrenia (12 control, 13 schizophrenia cases) [30]. In this study the authors reported that the increase in GAD67 protein occurred only in patients on antipsychotics at the time of death (n = 9) and suggested that this may reflect a compensatory mechanism in response to chronic antipsychotic medication. However, all cases in our cohort (n = 28) were on antipsychotics at the time of death, yet we report lower GAD at both mRNA and protein levels, suggesting that any possible increase in GAD67 by antipsychotic treatment is insufficient to restore GAD67 levels to control levels in our cohort. However, we also found that despite the overall decrease, GAD protein levels were positively correlated with duration of illness in schizophrenia patients, such that those who have been ill for longer have proportionally higher levels of GAD. Together with Schoonover et al. [30], this suggests that longer exposure to antipsychotics may recover a deficit in GAD levels in schizophrenia. However, we did not detect any significant relationships between lifetime, mean daily or last dose measures of antipsychotic exposure and GAD protein or mRNA levels in our cohort. In support of this, *GAD1* mRNA is unchanged in the cortex of monkeys treated chronically with antipsychotics [29, 79], and three schizophrenia cases with lower levels of cortical *GAD1* mRNA, who were not on antipsychotics, also showed lower GABA-related transcripts[79]. However, rodent studies indicate that antipsychotics sometimes have no impact, occasionally decrease and most often increase GABA indices [80–85]. In conclusion, while it is possible that antipsychotics could increase measures of inhibitory neurotransmission, they are not likely responsible for the decrease in GAD mRNA or protein levels that we report.

An important consideration when comparing the limited studies in the post-mortem midbrain is the anatomical level of the tissue. Schoonover et al. (2017) [30] utilised tissue from the caudal midbrain, Mabry et al. (2019) [78] used tissue from the middle of the rostrocaudal extent and our tissue was dissected from a rostral portion of the midbrain at the exit of the occulomotor nerve [17]; thus, it is possible that distinct changes in inhibitory action occur at different levels of the midbrain in people with schizophrenia. Taken together with the report of a reduction in inhibitory synapses in the midbrain in schizophrenia [78], we suggest that fewer inhibitory terminals may exist in the midbrain in conjunction with decreases in a key enzyme involved in GABA synthesis and vesicular GABA transporter, supporting the notion of less inhibitory control in the region of dopamine cell bodies in schizophrenia. Interestingly, in primates, rostral to caudal topographical organisation of basal ganglia circuitry controls different behaviours in parallel circuits [86]. Dopamine neurons in the rostral-ventral-medial SN project to the caudate head while those in the caudal-dorsal-lateral SN project to the caudate tail [87]. Caudate head-projecting DA neurons guide controlled behaviour, whereas caudate tail-projecting DA neurons guide automatic behaviour [86, 87]. Since GABAergic SNpr neurons send axon collaterals to adjacent DAergic SNpc neurons [21], this rostral-caudal organisation may also extend to inhibitory neuron control, suggesting that different changes in inhibitory control along this axis may impact distinct behaviours. We speculate that the potential loss of inhibitory control at a relatively rostral midbrain level would be most likely to impact more complex behaviour and striatal learning, both known to be altered at the behavioural and functional level in schizophrenia (e.g. [88, 89]).

In terms of which specific subcellular populations of GABAergic neurons are impacted, we found that *PV* mRNA was significantly lower in schizophrenia cases, at a magnitude of ~ 40%, akin to some reports in the cortex which range from 13% to > 50% [38, 39]. In contrast, and contrary to our hypothesis, *SST* mRNA was unchanged in our midbrain cohort, suggesting that there may be subtype specificity to the putative inhibitory neuron deficit and that this midbrain pathology is a notable distinction from the cortex where both PV + and SST + populations are impacted [7, 28, 32, 36–39]. Since PV + GABA-producing neurons with cell bodies located in the SNpr, project to the SNpc as well as other regions in the basal ganglia, the reduction in *PV* mRNA implicates the projection neurons of the SNpr in the potential GABAergic dysregulation in schizophrenia [40, 90]. Although it is a limitation of our study that we do not distinguish whether changes occur in a particular subpopulation of PV + neurons, our data suggests that GABAergic deficits in the midbrain may have implications for less overall inhibitory control throughout the basal ganglia in addition to a more local impact on the dopaminergic cell bodies forming the nigrostriatal pathway.

Our study showed that potential GABAergic dysregulation in the midbrain also extends to alterations in transcripts encoding postsynaptic GABAergic receptors. We showed the most substantial reduction (43.77%) in *GABRA1* mRNA, which encodes for the GABA_A_ α1 subunit that constitutes the α1β2γ2 receptor subtype, the most highly expressed GABA_A_ subtype in the brain [62, 91]. Although multiple subunits are identified in the midbrain [69], the subunit composition of GABA_A_ receptor subtypes specifically in the midbrain is not well established. However, the vast majority of α1 is detected on GAD67 + neurons within the SNpr, with low (7%) or no expression on pigmented (TH+) neurons of the SNpc [64, 65, 68, 69, 92]. Thus, reduction in GABA_A_ α1 may be occurring on both GABA and dopamine neurons in the midbrain in schizophrenia. However, the direct implications of lower levels of GABA_A_ receptor mRNAs for dopaminergic neurotransmission in the SN remain unclear. We found relationships between molecular markers of GABAergic and dopamine neurotransmission in the midbrain. Despite the substantial reduction in *GABRA1* mRNA in schizophrenia, positive correlations between *GABRA1* mRNA and dopaminergic (*TH* and *DAT)* mRNAs did not differ diagnostically, suggesting that, although there may be less α1 available in the midbrain in schizophrenia, the normal link between the α1 subunit and dopamine synthesis and transport remains intact. A limitation of our study is that we have not measured GABA_A_ beta or gamma subunits, which are highly expressed in (rat) midbrain [65, 69]. However, convergence of a reduction and dysregulation of multiple GABA_A_ α subunits is likely to contribute to schizophrenia pathophysiology in the midbrain.

We did measure gene expression of three other GABA_A_ receptor subunits and found consistent downregulation of α2, α3, and α5 in the midbrain in schizophrenia. The GABA_A_ α3 subunit is expressed on the dopaminergic neurons of the SNpc [63, 66], and we showed, similar to rat midbrain [69], 98% of dopaminergic neurons in the human midbrain have GABRA3 immunoreactivity in both controls and schizophrenia cases. The pattern of immunopositive α3 staining indicates the α3 subunit likely contributes to the composition of cell surface GABA_A_ receptors on dopamine neurons. To our knowledge, the α3 subunit has not been studied before in schizophrenia, though an animal model suggests a direct link between the absence of this receptor and a hyperdopaminergic phenotype [93], supporting the notion that an overall reduction in GABA_A_ receptors containing this subunit in the SN may enable disinhibition of dopaminergic neurons and contribute to nigrostiatal dopamine dysregulation. A caveat is that we did not detect significantly lower GABRA3 protein in the midbrain homogenates. However, this may be due to Western blotting being a less sensitive method with greater inherent variance. In addition, we found that *GABRA3* and *GABRA2* mRNAs were highly positively correlated with *TH* and *DAT* mRNAs in schizophrenia cases but not controls. As such, despite overall reductions in transcripts, positive correlations suggest that levels of these DA and GABA_A_ receptor transcripts are changed in concert in schizophrenia. We can speculate that higher TH levels would lead to more DA synthesis and correspondingly higher GABA_A_ receptor levels may therefore reflect a compensatory response to attenuate DA neurotransmission. Conversely, higher DAT levels in concert with increased inhibition would potentially yield an opposing effect on DA neurotransmission, as more DAT would mean a greater propensity to terminate dopamine neurotransmission. Thus, in schizophrenia midbrain, altered local interactions, which are established as crucially important to the functioning of the SN, between SNpr GABAergic neurons and DAergic neurons [21] may result in a functional change in the local inhibitory control of dopamine neurotransmission in the disease state.

The decrease in *GABRA3* mRNA was the only change that was consistent with our hypothesis that GABA deficits would be most pronounced in cases with a high inflammatory biotype. We previously established that the midbrain is a site of convergence of macrophage presence, microglial activation, and enhanced activity of the complement cascade [54, 55], and the convergence of these inflammatory processes may lead to disruption of neurotransmitter systems within the midbrain. It is possible that a global deficit in GABAergic neurotransmission and a heightened state of neuroinflammation in the midbrain coalesce to impact the GABRA3-expressing dopaminergic neurons of the nigrostriatal pathway, although mechanistic studies are needed to test this hypothesis.

We also detected significant reductions in *GABRA2* and *GABRA5* transcripts, although previous studies of their cellular location are contradictory. In the mouse brain, *GABRA5* mRNA is not detected but *GABRA2* mRNA is found in 99.5% of TH + cells in the SNpc [69], yet only weak immunohistochemical labelling of the α2 subunit is identified in the rat SNpc [65]. Previous studies in human midbrain failed to identify either mRNA, using in situ hybridisation, or protein measurements for the α2 subunit [66]. However, we detected *GABRA2* mRNA in the human midbrain (albeit at lower levels than *GABRA1* mRNA) with RT-qPCR which is inherently more sensitive to amplifying low abundant transcripts than in situ hybridisation. We previously used the same primer and probe set to amplify *GABRA2* transcripts in the human cortex [25], and in agreement with previous studies [69], we have also detected *GABRA2* transcripts in the rat substantia nigra (Additional file 1: Fig. S3). In addition, we showed a diagnostic difference in the relationships between *GABRA2* (but not *GABRA5)* mRNA and *TH* and *DAT* mRNAs, suggesting that our *GABRA2* mRNA measures are bona fide. We suggest that the tandem disruptions in the relationships between dopamine transcripts and GABA receptor α subunits may reveal the subunit composition of receptor subtypes that are most dysregulated in the midbrain in schizophrenia. Further studies are required to characterise cell-specific GABA_A_ receptor subtypes in the human midbrain and how these may be altered in the dopamine neurons in schizophrenia.

The reduction in GABA_A_ receptors in the brain in schizophrenia has formed the basis of GABA_A_ receptor agonist (benzodiazepines) clinical trials; however, they have produced inconsistent results in schizophrenia patients. Our study supports animal studies that indicate that harnessing the ability to target GABA receptor subtypes based on their cellular specificity could bring about symptomatic attenuation in a more targeted manner [93–95], and thus may yet hold therapeutic potential. Our findings extend the body of evidence implicating GABAergic dysfunction in the pathophysiology of schizophrenia, to the main dopaminergic locus of the brain. We localised GABAergic dysregulation to neuronal populations that have cell bodies within the SN, which may have implications for both the circuitry of the basal ganglia and for nigrostriatal dopaminergic neurotransmission in schizophrenia. Furthering our understanding of the underlying neurobiology within the midbrain in schizophrenia provides impetus for exploring GABA-related treatments specifically targeting this brain region.

## Supplementary Information

**Additional file 1.** Supplementary methods, figures and tables.

## Data Availability

The datasets analysed during the current study are available from the corresponding author on reasonable request.
