# Supplementary material for: Reductions in midbrain GABAergic and dopamine neuron markers are linked in schizophrenia
Source: Mol Brain. 2021 Jun 26;14:96. doi: 10.1186/s13041-021-00805-7 (PMC8235806; doi:10.1186/s13041-021-00805-7)
Supplement: Supplementary file 1 — Additional file 1. Supplementary methods, figures and tables. [file 13041_2021_805_MOESM1_ESM.docx]

**Reductions in midbrain GABAergic and dopamine neuron markers are linked in schizophrenia – Supplementary Material**

Tertia D. Purves-Tyson^1,2^*^#^, Amelia M. Brown^1#^, Christin Weissleder^1^, Debora A. Rothmond^1^ and Cynthia Shannon Weickert^1,2,3^*

^1^ Schizophrenia Research Laboratory, Neuroscience Research Australia, Sydney, NSW 2031, Australia

^2^ School of Psychiatry, Faculty of Medicine, University of New South Wales, Sydney, NSW 2052, Australia

^3^ Department of Neuroscience & Physiology, Upstate Medical University, Syracuse, New York 13210, USA

# Joint First Authors

**Joint Corresponding Authors:*

Tertia Deborah Purves-Tyson, PhD

Neuroscience Research Australia

Schizophrenia Research Laboratory

139 Barker Street, Margarete Ainsworth Building, Level 5

Randwick NSW 2031 Australia

T: +61 2 9399 1751

F: +61 2 9399 1005

E: t.purves-tyson@neura.edu.au

Cynthia Shannon Weickert, Ph.D.

Neuroscience Research Australia

Schizophrenia Research Laboratory

139 Barker Street, Margarete Ainsworth Building, Level 5

Randwick NSW 2031 Australia

T: +61 2 9399 1717

F: +61 2 9399 1005

E: [cyndi@neura.edu.au](mailto:cyndi@neura.edu.au)

**SUPPLEMENTARY METHODS**

***Post-Mortem Midbrain Tissue and Cohort Demographics Additional Information***

Freezer storage time

After dissection, brains were hemisected, one hemisphere fresh frozen and stored at -80°C until processed by sectioning. Tissue was cryostat-sectioned at -20°C, alternating between 60 μm sections (stored between wax paper at -80°C), and 14μm sections (captured on gelatinised glass slides and stored at -80°C). The average freezer storage time in months (from dissection to sectioning) for controls is 89±57.44 months and for schizophrenia cases is 69.4±42.45. There was no significant difference in freezer storage time between controls and schizophrenia cases (*p* = 0.152).

Antemortem factors

As described previously in [1], all schizophrenia patients received antipsychotic medication. Throughout their illness 6–6 (mRNA–protein cohort) patients received first-generation antipsychotics only, 11–8 patients had predominantly first generation, 5–6 patients received equal first-generation and second-generation antipsychotics, 5–5 patients received predominantly second-generation antipsychotics and 1–1 received second generation only. As clozapine is generally only recommended after at least two trials of other antipsychotics have failed to have a benefit, clozapine treatment at time of death (n = 7-7) was used as an indicator of possible treatment resistance versus all other antipsychotics at time of death (n = 21-19). Antipsychotic medication was converted to chlorpromazine (CPZ) equivalents (lifetime, daily and last dose). The schizophrenia cases were diagnosed with either more prevalent positive symptoms (n = 22) or more prevalent negative symptoms (n = 7) with one unknown. Toxicology screening of control cases at time of post-mortem revealed nothing (n=10), diazepam (n = 1), cannabis (n = 1), paracetamol (n = 7), codeine (n = 3) and blood pressure medication (n = 2), and some (n = 5) had no toxicology screen with cardiac failure recorded as cause of death. Toxicology screening of schizophrenia cases revealed no comorbid substance use (n = 13), methadone and diazepam (n = 1), diazepam and insulin (n = 1), diazepam, pethidine, codeine and paracetamol (n = 1), codeine, diazepam and morphine (n = 1), paracetamol only (n = 4), a non-steroidal anti-inflammatory (n = 1), morphine (n = 1) codeine (n = 1) and ibuprofen (n = 1) and screening not available (n = 5). Alcohol consumption at time of death in controls and schizophrenia cases was as follows: nil (n=1–0), <20 g per day (n = 17–22), 20–50 g per day (n = 6–3), 480 g per day (n = 1–3) and unknown (n = 4–2). A history of depression symptoms during lifetime was identified in eight schizophrenia cases (six were treated with serotonin-selective reuptake inhibitors (SSRIs) and two with tricyclic antidepressants) and in one control (treated with SSRIs).

**
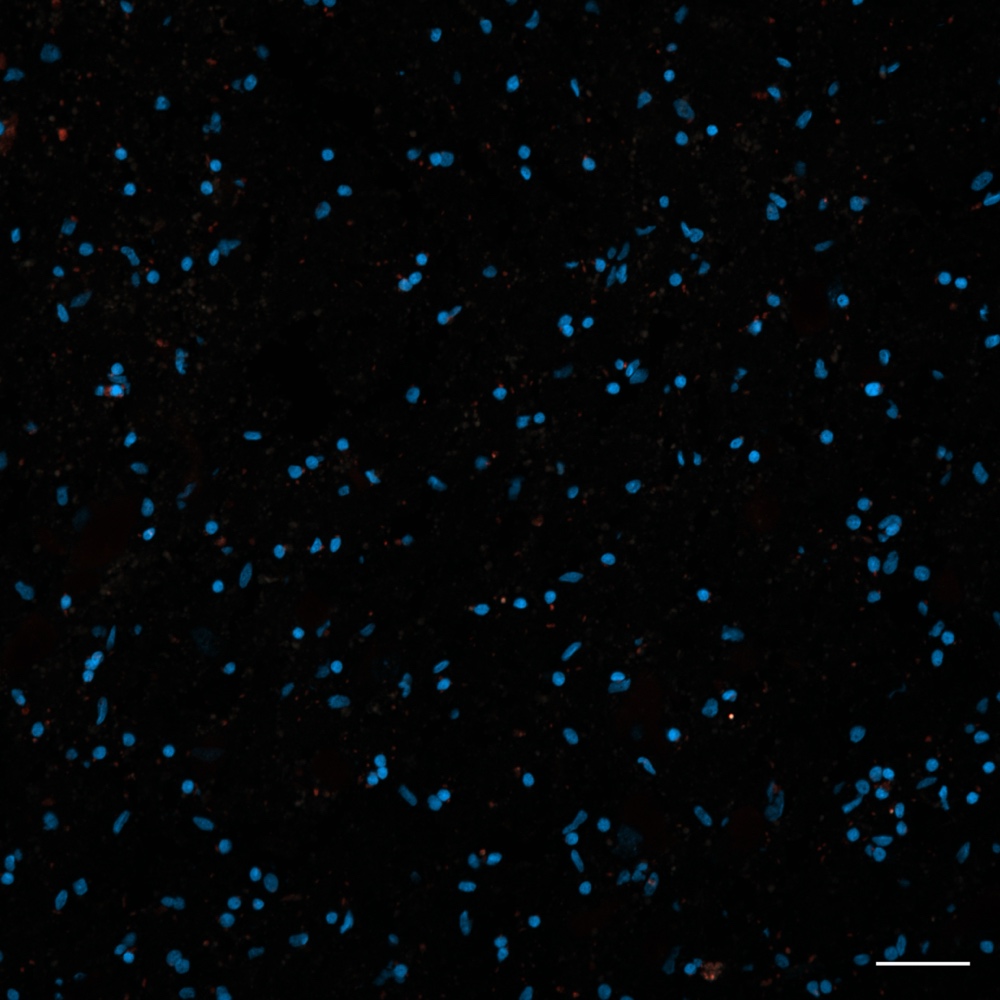
SUPPLEMENTARY FIGURES**

**Supplementary Figure 1.** A slide incubated with no primary antibodies but both secondary antibodies to visualise non-specific secondary antibody binding or auto-fluorescence shows no fluorescent signal and confirms the specificity of the fluorescent signal when primary antibodies are present. Nuclei are stained blue with DAPI. Scale bar = 50 µm.

**Supplementary Figure 2.** Full Western blots of human midbrain homogenates showing a single GAD protein band at ~66 kDA (A, left) detected with a rabbit anti-glutamate decarboxylase 65/67 antibody (1:1000, AB1511, Sigma-Aldrich) and a prominent GABRA3 band at ~55 kDA (B, left) detected with a rabbit anti-GABA Receptor α3 antibody (1:1000, ab224214, Abcam, Melbourne, VIC, AUS). GAD immunoblot was not stripped prior to re-probing with a mouse anti-β-actin antibody (1:5000, MAB1501, Merck-Millipore), whilst this GABRA3 immunoblot was stripped with 2 x 15 min washes in 0.025M glycine, 1.5% SDS buffer prior to re-probing with the same β-actin antibody. A β-actin band was detected at ~42 kDa. MWM, molecular weight marker, (Precision Plus Protein Standards, Biorad #161-0374). Red arrow denotes the 50 kDA molecular weight marker. The hashed block shows the bands depicted in Figures 1 and 4 in the manuscript. Marker in the centre of the blots was not used to determine molecular weights.

**Supplementary Figure 3.** (A) *GABRA2* mRNA detected in substantia nigra (SN) of normal male and female adult (postnatal day 86-87) Wistar rats (n = 19) using the same high-throughput RT-qPCR method, and a rat specific TaqMan assay # Rn01413643_m1, used to detect *GABRA2* transcripts in the human midbrain. (B) *GABRA2* mRNA 7-point standard curve generated with a 3-fold serial dilution (undiluted, 1:3, 1:9, 1:27, 1:81, 1:243, 1:729) of pooled cDNA from 10 rat SN. (C) Amplification curves of rat SN cDNA obtained from Fluidigm Real-Time PCR Analysis software (version 4.5.2). The amplification curves include all treatment/diagnostic groups within the study, not just the control group reported in (A).

**SUPPLEMENTARY TABLES**

**Supplementary Table 1.** List of probes used for RT-qPCR.

| Gene of Interest | TaqMan Accession Number |
| --- | --- |
| GABRA1 | Hs00975293_m1 |
| GABRA2 | Hs00941404_m1 |
| GABRA3 | Hs00968132_m1 |
| GABRA5 | Hs00894847_m1 |
| GAD1 | Hs01065893_m1 |
| PV | Hs00161045_m1 |
| SST | Hs00356144_m1 |
| VGAT | Hs00369773_m1 |
| ACTB | Hs99999903_m1 |
| UBC | Hs00824723_m1 |
| TBP | Hs00427620_m1 |
| GAPDH | Hs99999905_m1 |

**Supplementary Table 2.** Correlations between GABAergic gene and protein expression and post-mortem variables.

| Gene of interest | Variable | N | Correlation coefficient | p |
| --- | --- | --- | --- | --- |
| GABRA1 mRNA | RIN  pH  PMI  Age | 54  54  54  54 | **0.562**  **0.625**  0.086  0.005 | **<0.001*****  **<0.001*****  0.537  0.969 |
| GABRA2 mRNA | RIN  pH  PMI  Age | 52  52  52  52 | 0.218  **0.372**  0.153  -0.178 | 0.121  **0.007****  0.278  0.207 |
| GABRA3 mRNA | RIN  pH  PMI  Age | 54  54  54  54 | **0.382**  **0.475**  0.172  0.035 | **0.004****  **<0.0001******  0.212  0.800 |
| GABRA3 protein | pH  PMI  Age | 47  47  47 | -0.102  -0.210  -0.090 | 0.494  0.156  0.547 |
| GABRA5 mRNA | RIN  pH  PMI  Age | 53  53  53  53 | **0.353**  **0.288**  0.109  0.270 | **0.010***  **0.037***  0.435  0.051 |
| GAD1 mRNA | RIN  pH  PMI  Age | 53  53  53  53 | **0.375**  **0.579**  0.140  -0.020 | **0.006****  **0.002****  0.318  0.889 |
| GAD protein | pH  PMI  Age | 52  52  52 | 0.154  -0.223  -0.086 | 0.277  0.113  0.543 |
| PV mRNA | RIN  pH  PMI  Age | 54  54  54  54 | **0.277**  **0.445**  **0.307**  0.112 | **0.043***  **<0.001*****  **0.024***  0.420 |
| SST mRNA | RIN  pH  PMI  Age | 52  52  52  52 | 0.236  0.201  0.113  -0.239 | 0.092  0.153  0.426  0.088 |
| VGAT mRNA | RIN  pH  PMI  Age | 55  55  55  55 | **0.473**  **0.620**  0.144  0.009 | **<0.001*****  **<0.001*****  0.294  0.945 |

**p* < 0.05, ***p* < 0.01, ****p* < 0.001, *****p* < 0.0001.

**Supplementary Table 3.** Correlations between measures of antipsychotic use (lifetime, mean daily and last dose chlorpromazine equivalents) and illness duration and GABAergic genes of interest.

| Gene of interest | Variable | N | Correlation  coefficient | *p* |
| --- | --- | --- | --- | --- |
| GABRA1 mRNA | Lifetime  Mean daily  Last dose  Illness duration | 21  21  27  27 | 0.0005  0.006  0.252  -0.108 | 0.982  0.980  0.204  0.591 |
| GABRA2 mRNA | Lifetime  Mean daily  Last dose  Illness duration | 20  20  26  26 | -0.255  -0.370  0.033  -0.210 | 0.278  0.108  0.873  0.303 |
| GABRA3 mRNA | Lifetime  Mean daily  Last dose  Illness duration | 21  21  27  27 | -0.284  -0.347  -0.164  -0.122 | 0.212  0.123  0.412  0.544 |
| GABRA3 protein | Lifetime  Mean daily  Last dose  Illness duration | 15  15  20  19 | -0.111  0.014  -0.252  0.116 | 0.694  0.960  0.283  0.636 |
| GABRA5 mRNA | Lifetime  Mean daily  Last dose  Illness duration | 21  21  27  27 | -0.240  -0.164  0.246  -0.167 | 0.295  0.478  0.216  0.404 |
| GAD1 mRNA | Lifetime  Mean daily  Last dose  Illness duration | 21  21  27  27 | -0.205  -0.186  0.059  -0.047 | 0.372  0.420  0.769  0.815 |
| GAD protein | Lifetime  Mean daily  Last dose  Illness duration | 19  19  25  24 | 0.219  0.079  -0.267  **0.490** | 0.367  0.748  0.197  **0.015*** |
| PV mRNA | Lifetime  Mean daily  Last dose  Illness duration | 22  22  26  26 | -0.091  -0.188  0.175  -0.062 | 0.685  0.402  0.391  0.762 |
| SST mRNA | Lifetime  Mean daily  Last dose  Illness duration | 22  22  27  27 | -0.234  -0.179  -0.022  0.107 | 0.294  0.425  0.912  0.603 |
| VGAT mRNA | Lifetime  Mean daily  Last dose  Illness duration | 21  21  27  27 | -0.127  -0.116  0.063  -0.105 | 0.582  0.618  0.754  0.601 |

**Supplementary Table 4.** Analysis of clinical and ante-mortem variables for each GABAergic transcript measured. Not all variables were available for all cases, hence the variable group numbers. Significant results are bolded and italicised * *p* < 0.05. ^schizophrenia cases only, ^&^controls and schizophrenia cases included.

| Variable | GABRA1 | GABRA2 | GABRA3 | GABRA5 | VGAT | SST | GAD1 | PV |
| --- | --- | --- | --- | --- | --- | --- | --- | --- |
| Antipsychotic type (first generation, second generation or both, (6,17,5)^ | F(2,24)=0.351,  p=0.707 | F(2,23)=0.377,  p=0.690 | F(2,24)=0.241, p=0.788 | F(2,24)=1.407, p=0.264 | F(2,24)=1.404,  p=0.265 | F(2,24)=1.699, p=0.204 | F(2,24)=0.846, p=0.441 | F(2,23)=1.829,  p=0.183 |
| Positive vs negative symptoms  (18,7)^ | t(22)=-0.577, p = 0.570 | t(21)= -1.576,  p=0.130 | t(22)=-1.547, p=0.136 | t(22)=-0.274, p=0.786 | t(22)=-0.778, p=0.445 | t(22)=-0.593, p=0.559 | t(22)=-1.035, p=0.312 | t(21)=-0.131, p=0.897 |
| Clozapine at time of death (treatment resistance) vs other antipsychotics (n=7, 21)^ | t(16.36)=-0.496,  p=0.626 | t(20.11)=1.023,  p=0.319 | t(25)=-0.712, p=0.483 | t(25)=-0.819, p=0.421 | t(25)=-0.406, p=0.688 | t(25)=0.325, p=0.748 | t(25)=0.680, p=0.503 | t(24)=-0.010, p=0.992 |
| Suicide completers vs non suicide death (7/21)^ | t(25)=-0.821, p=0.419 | t(24)=-1.305, p=0.204 | t(25)=-1.241, p=0.226 | t(25)=-0.460, p=0.650 | t(25)=-1.624, p=0.115 | t(25)=-1.569, p=0.129 | ***t(25)=-2.071, p=0.049**** | t(24)=-1.156, p=0.259 |
| Agonal state (excellent/good/unknown 35/18/3)^&^ | F(1,47)=0.076, p=0.784 | F(1,45)=3.086, p=0.086 | F(1,47)=0.593, p=0.445 | F(1,46)=0.686, p=0.412 | F(1,49)=0.122, p=0.728 | F(1,45)=0.026, p=0.873 | F(1,47)=0.002, p=0.968 | F(1,47)=0.049, p=0.825 |
| Smoking at death (yes/no/unknown, 26/18/12)^&^ | F(1,38)=0.438, p=0.512 | F(1,36)=0.013, p=0.910 | F(1,38)=1.030, p=0.317 | F(1,37)=0.336, p=0.565 | F(1,39)=1.022, p=0.318 | F(1,38)=0.499, p=0.484 | F(1,37)=0.001, p=0.977 | F(1,39)=0.007, p=0.932 |
| Smoking lifetime  (yes/no/unknown, 29/15/12)^&^ | F(1,38)=0.423, p=0.520 | F(1,36)=0.002, p=0.968 | F(1,38)=0.925, p=0.342 | F(1,37)=0.148, p=0.703 | F(1,39)=0.942, p=0.338 | F(1,38)=0.062, p =0.805 | F(1,37)=0.003, p=0.958 | F(1,39)=0.061, p=0.806 |
| Symptoms of depression during lifetime vs none (8,19)^ | t(22)=-0.577, p=0.570 | t(21)=-1.576, p=0.130 | t(22)=-1.547, p=0.136 | t(22)=-0.274, p=0.786 | t(22)=-0.778, p=0.445 | t(22)=-0.593, p=0.559 | t(22)=-1.035, p=0.312 | t(21)=-0.131, p=0.897 |

**Supplementary References**

1. Purves-Tyson, T.D., S.J. Owens, D.A. Rothmond, G.M. Halliday, K.L. Double, J. Stevens, T. McCrossin, and C. Shannon Weickert: **Putative presynaptic dopamine dysregulation in schizophrenia is supported by molecular evidence from post-mortem human midbrain.** Transl Psychiatry 2017. **7**(1): p. e1003.
